# High-density linkage map and QTL analyses for fruit quality traits in the wild blueberry relative *Vaccinium stamineum*

**DOI:** 10.1093/g3journal/jkaf263

**Published:** 2025-11-12

**Authors:** Gabriel O Matsumoto, Alexandria Garcia, Juliana Benevenuto, Patricio R Munoz

**Affiliations:** Blueberry Breeding and Genomics Lab, Horticultural Sciences Department, University of Florida, Gainesville, FL 32611, United States; Blueberry Breeding and Genomics Lab, Horticultural Sciences Department, University of Florida, Gainesville, FL 32611, United States; Blueberry Breeding and Genomics Lab, Horticultural Sciences Department, University of Florida, Gainesville, FL 32611, United States; Blueberry Breeding and Genomics Lab, Horticultural Sciences Department, University of Florida, Gainesville, FL 32611, United States

**Keywords:** deerberry, breeding, SNP, quantitative trait loci, sweetness, acidity

## Abstract

The wild blueberry relative *Vaccinium stamineum* offers a rich source of diversity for expanding the genetic pool of cultivated blueberries, and it has also potential for breeding as a crop on its own. Understanding the genetic architecture of fruit quality traits in this wild species can facilitate and speed up future breeding efforts for introgression and de novo domestication. Therefore, in this study, we developed a biparental population of 147 progenies from *V. stamineum*, which were phenotyped and genotyped for quantitative trait loci (QTL) mapping. Phenotypic data for acidity [total titratable acidity {TTA} and pH] and sweetness (soluble solids content) were collected over 3 yr. Genotypic data were obtained through targeted sequencing using 6,000 probes developed for blueberry. A linkage map was crafted containing a total of 3,797 markers spanning a cumulative distance of 1,801 cM across 12 linkage groups. Composite interval mapping revealed a total of 20 significant QTL considering the 3 traits and years of evaluation. Two consistent overlapping QTL intervals across 2 yr were found for TTA and soluble solids in linkage groups 8 and 9, respectively. We also found a QTL for TTA that has been previously reported for cultivated blueberry. Low to moderate heritability was observed, indicating the complex genetic architecture of these traits. Overall, the newly developed high-density genetic map provides a valuable resource for trait mapping efforts in this species, and the QTL identified for fruit quality can guide future molecular breeding strategies.

## Introduction

*Vaccinium stamineum*, commonly known as deerberry, is a highly polymorphic species native to North America, with a widespread geographic distribution ranging from isolated populations in Mexico to Canada and from Texas to the eastern United States (Vander Kloet 1988). This species belongs to same genus of cultivated berry crops, such as highbush blueberry (*Vaccinium corymbosum*) and cranberry (*Vaccinium macrocarpon*). Although *V. stamineum* is classified into a different section of the genus (*Polycodium* section), it formed the (Hill 2002; Becker et al. 2024) closest clade to blueberry species from the *Cyanococcus* section (Hill 2002; Becker et al. 2024). Viable intersectional crosses of *V. stamineum* and *V. corymbosum* have been achieved (Lyrene 2018, 2021). However, given the differences in ploidy levels, with *V. corymbosum* being autotetraploid and *V. stamineum* being diploid, the identification of accessions that produce unreduced gametes or employing chromosome doubling agents is required to overcome a strong triploid block (Lyrene 2018, 2021).

The ability to thrive in well-drained soils with limited annual rainfall and tolerance to acidic soil conditions is one of the several desirable traits that can be introgressed from *V. stamineum* (Hill 2002; Lyrene 2018). Additionally, *V. stamineum* possesses a unique open-flower morphology that facilitates cross-pollination and naturally large fruits when compared to other wild *Vaccinium* species (Vander Kloet 1988). Its berries can accumulate anthocyanin in the berry pulp and contain a high content of soluble solids and unique flavor profiles (Ballington et al. 1984; Vander Kloet 1988; Horvat et al. 1996), further enhancing its appeal as a potential fruit crop on its own. However, despite its potential, *V. stamineum* remains largely undomesticated, but the University of Florida is actively working on introgressing it into the blueberry breeding program.

From a cultivation perspective, several challenges need to be addressed to advance the utilization of *V. stamineum* in breeding programs. One major limitation is its tendency to shatter ripe berries (Vander Kloet 1988), which complicates harvest and production. Additionally, the species exhibits significant variation in fruit quality traits, especially derived from fruit skin bitterness. Therefore, prior to any breeding efforts, a germplasm selection aiming for consistent and palatable traits is necessary (Ballington 2008). Addressing some of the key hindrances for the usage of *V. stamineum* can be taken as an important first step toward the utilization of this species in breeding programs. To aid in this task, the use of genomic tools has been proven to be an effective strategy for more accurate selection and shortening of breeding cycles (Ferrão et al. 2020; Kumar et al. 2024). By leveraging molecular markers associated with traits in wild species, breeders can track the introgressed regions from wild relatives across generations, ensuring that beneficial alleles are retained while minimizing the transfer of undesirable traits.

To this end, this study focused on generating the first genetic map for *V. stamineum* using a biparental population. This genetic map was then used as the foundation for QTL mapping to unravel the genetic architecture of key fruit quality parameters [total titratable acidity {TTA}, pH, and soluble sugar content], shedding light onto the integration of *V. stamineum* in breeding pipelines.

## Material and methods

### Plant material

A biparental population of *V. stamineum* was generated from a cross between the unpatented accessions named “AP3” and “Crispsweet.” These genotypes are part of the germplasm collection from the blueberry breeding program at the University of Florida. In 2017, flowers of “AP3” (female parent) were hand pollinated with pollen from “Crispsweet” in a greenhouse. A total of 178 progenies were grown in 40-L pots in Gainesville, FL, United States. The accession “AP3” produces small berries, typically 9 to 10 mm in diameter, with red-colored pulp and a bitter peel. In contrast, “Crispsweet” produces larger berries, ranging from 11 to 14 mm in diameter, with green to pale-colored pulp and little to no bitterness in the peel. The berries of “Crispsweet” are sweet and exhibit a crisp texture. Both accessions, “AP3” and “Crispsweet,” form an upright bush similar to the plant architecture of *V. corymbosum*.

### DNA sequencing and probe alignment

Genomic DNA was extracted from the leaves of the *V. stamineum* biparental population, followed by library preparation. Sequencing was performed by RAPiD Genomics (Gainesville, FL, United States). Capture-seq, a targeted sequencing approach, was used to genotype this population. In this strategy, 6,000 probes of 120-mer originally designed based on the *Vaccinium caesariense* “W85-20” reference genome were used (Ferrão et al. 2018; Benevenuto et al. 2019). Sequencing was performed in the Illumina HiSeq 2000 platform using 150 cycle paired-end runs. Initial processing of the raw reads included demultiplexing, barcode removal, and trimming the 3′ end by removing bases with quality scores lower than 20. In addition, reads with more than 10% of the bases with a quality score below 20 were also removed using Fastx Toolkit v.0.0.14. Given that the probes were designed based on a different species, probe positions were remapped in the *V. stamineum* reference genome (Matsumoto et al. 2025). Thus, the 6,000 Capture-seq probe sequences were aligned to the assembled masked genome using the command line NCBI blastn/2.15.0 function with the options -evalue 0.00001 -perc_identity 95 to retain highly similar hits while still allowing for some mismatches. The blast output was also filtered to remove multiple mapping and short query coverage. Probe coordinates in the *V. stamineum* genome were then obtained for targeted variant calling.

### Variant calling

First, bwa/0.7.17 (Li 2013) was used to map the trimmed Capture-seq reads to the *V. stamineum* reference genome (Matsumoto et al. 2025). Then, samtools/1.15 (Danecek et al. 2021) converted the SAMfiles to a sorted BAM format as input for variant calling using freebayes/1.3.2 (Garrison and Marth 2012). The probe coordinates in the *V. stamineum* genome were used as targets for variant calling. Single nucleotide polymorphisms (SNPs) were filtered using vcftools/0.1.16 (Danecek et al. 2011) with parameters set for minimum mapping quality of 30, minor allele frequency of 0.20 given that this is a F1 diploid biparental population, maximum missing data of 10%, minimum sequencing depth of 12 reads, and biallelic alleles only.

The variants identified in this population were used to generate a principal component analysis (PCA) to explain the genetic variance present among individuals, using the *SNPRelate* R package (Zheng et al. 2012). All individuals that did not fall within the expected parental cluster were then removed from the subsequent analysis.

### Linkage mapping

The construction of a genetic map was carried out using the onemap R package (Margarido et al. 2007; Schiffthaler et al. 2017). Bins of redundant markers that carry the same genotypic information were removed from the dataset to simplify the data analysis. In addition, segregation distortion in the marker SNP dataset was tested to filter out markers deviating from the expected segregation, and the *suggest_lod()* function was used to identify a suggested LOD score to determine statistical significance of the 2-point tests for linkage between pairwise marker comparisons. The remaining markers were separated into groups based on the *V. stamineum* chromosomes. Therefore, each linkage group (LG) was built independently with the *create_probs* function assuming a global error of 0.05. In each LG, the marker initial ordering was estimated taking their genomic positions into consideration. The marker ordering was estimated by batches with an overlap of 30 markers per batch. Fine adjustments in the LG were done by removal or reorganization of markers, as needed, until a map with minimal overall inflation and cohesion between neighboring markers was achieved.

To verify the quality of the linkage map, the recombination fraction (rf) matrices were generated for each LG for a pairwise assessment of the recombination between markers.

### Phenotyping

Branches from each plant were bagged with polyethylene mesh bags when berries were at the fully expanded green stage to avoid loss by fruit shattering. Then, ripe berries were harvested when the pedicels changed from green to red, as an indicator of full maturation. A total of 25 berries were used in the fruit quality pipeline, including soluble solids (hereafter referred to as °Brix), TTA, and pH. Upon harvest, ripe berries were kept at 5 °C until evaluation, which occurred within the first 48 h after harvesting. Phenotypic data were collected during June/July during the years of 2021, 2022, and 2023.

The fruit quality data were generated, first, by blending the berries into a puree and centrifuging for 10 min at 10,000 *g* and 32 °C. The juice was filtered using a cheesecloth and captured into Falcon tubes using a funnel. This juice was assessed for soluble solids using a refractometer (Atago, Tokyo, Japan), while both TTA and pH measurements were calculated using a pH meter (Mettler Toledo G10S Titrator, Columbus, OH, United States). Total titratable acidity measurements were carried out in a blend of a 3 mL of the berry juice and 50 mL of double distilled water, using 0.1N NaOH as the base.

Correlation analyses were conducted among the samples with complete data across all years. Specifically, an all-vs-all comparison was performed using Spearman's correlation analysis implemented in the *corrplot* R package.

### QTL mapping and statistical analysis

The phenotypic data were combined with the genetic map to conduct QTL mapping. Composite interval mapping (CIM) within the R package *fullsibQTL* was then employed to scan the genome for quantitative trait loci (QTL) (Amadeu et al. 2020 ). The Akaike information criterion (AIC) was used to refine the models, followed by the characterization of the QTL windows for each trait individually (Gazaffi et al. 2014; Amadeu et al. 2020). A maximum of 10 cofactors were used to refine the precision of QTL identification. Finally, the threshold of QTL detection based on the LOD for each trait was calculated based on 1,000 permutations, and a QTL was considered significant at a α = 0.05. To colocalize significant QTL identified in this study compared to QTL previously identified in the literature, genome-wide alignments between *V. stamineum* (Matsumoto et al. 2025) against *V. corymbosum* “Draper” (Colle et al. 2019) and *V. caesariense “*W85-20” (Mengist et al. 2022a) were performed using the minimap2/2.28 software (Li 2018).

Variance components were estimated with a restricted maximum likelihood (REML) using ASReml version 4.2 (Butler et al. 2023 ). The following univariate mixed model was used to analyze °Brix, TTA, and pH:


$$y\;=\;X\beta +Z_{1}u\;+\;Z_{2}gxe\;+\;\varepsilon$$

where *β* is the vector of year as a fixed effect, *u* is the random genetic effect for the genotypes modeled using the genomic relationship matrix G (u∼*N*(0,Gσ_u_^2^​)), *gxe* is the random genotype × year interaction effect also modulated by G (gxe∼*N*(0,Gσ_gxe_^2^​)), and *ε* is the random residual effect. *X*, *Z_1_*, and *Z_2_* are the incidence matrices for the response variable *y*. The G matrix was calculated with the AGHmatrix package in R (Amadeu et al. 2023) using the full dataset of SNPs identified in the variant calling process. The trait heritability was calculated by *h^2^ = σ_u_^2^  _/ (_σ_u_^2^* + *σ_gxe_^2^* + *σ_ε_^2^).* To assess the significance of the genotype-by-year interaction, a likelihood ratio test (LRT) was performed by comparing the full model (as above) to a reduced model excluding the random genotype-by-year interaction term. Finally, Wald test was performed for year assuming the year as the fixed effect in the mixed model.

## Results

### Probe alignment and variant calling

**Fig. 1.**
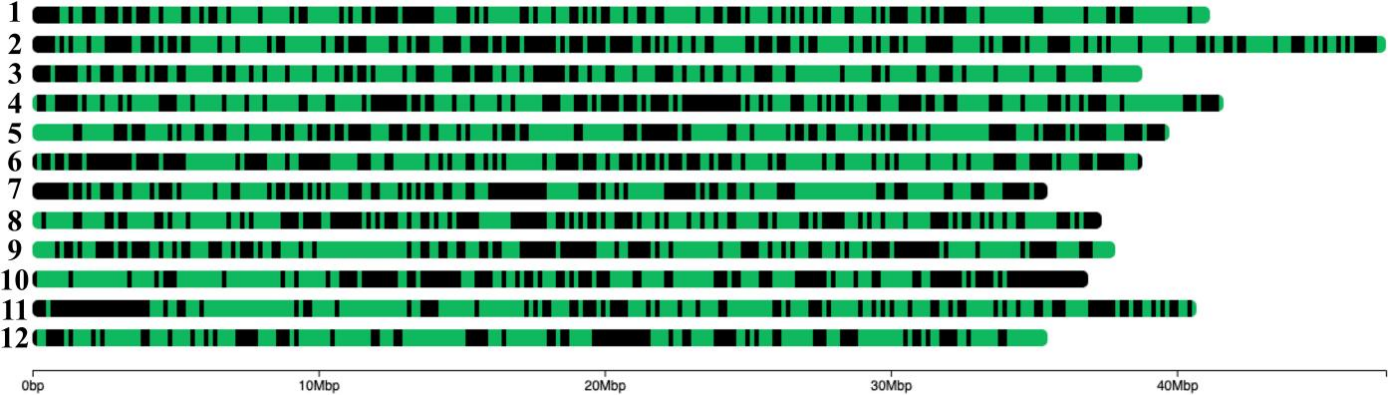
Blueberry target-genotyping probes mapped to the *V. stamineum* reference genome. Green color represents bins of 50 Kb that have at least 1 probe covering that region, while black color represents bins that did not have any probes mapped to that region.

The probes used for genotyping were designed based on the *V. caesariense* “W85-20” reference genome (Bian et al. 2014; Gupta et al. 2015). These probes were mapped against the primary haplotype of the *V. stamineum* “AP3” reference genome using BLASTN searches. A total of 4,245 probes out of the initial 6,000 exhibited corresponding matches on the *V. stamineum* reference genome, and consequently, the regions of the genome covered by these probes were targeted for subsequent analysis. Despite the probes being designed for a different species, the only genomic regions that appear to be underrepresented are the end of chromosome 10 and the start of chromosome 11, while most of the other chromosomes seem to be well covered by the probes (Fig. 1).

Following this step, a total of 14,191 SNPs were detected across the biparental population. This set of variants was further filtered based on the parameters previously mentioned, resulting in a total of 4,494 SNPs used for genetic analysis. In addition, it was possible to identify possible contaminants in the population through the PCA with the SNP dataset. The final population contained 147 individuals that were used in the following analyses (Supplementary Fig. 1).

### Linkage mapping

First, the marker data were binned to remove redundant information, reducing the complexity of map construction. The remaining markers were assessed for segregation distortion, and only those following specific patterns were considered for the final map (Supplementary Fig. 2). Specifically, markers of types D1.10 and D2.15 that exhibit a 1:1 segregation ratio with 1 parental line heterozygous and the other homozygous at a given locus (e.g. “ab × aa” or “aa × ab”) were included. Additionally, markers of type B3.7 with a double heterozygous locus (e.g. “ab × ab’) that follow a 1:2:1 segregation ratio were also considered for map construction (Supplementary Fig. 2). All markers that did not follow the expected segregation ratio were removed from the analysis. The resulting set of markers for linkage mapping consisted of 3,227 SNPs. These markers were then grouped based on the chromosomes they belonged to, and the genetic map was built independently for each LG.

**Fig. 2.**
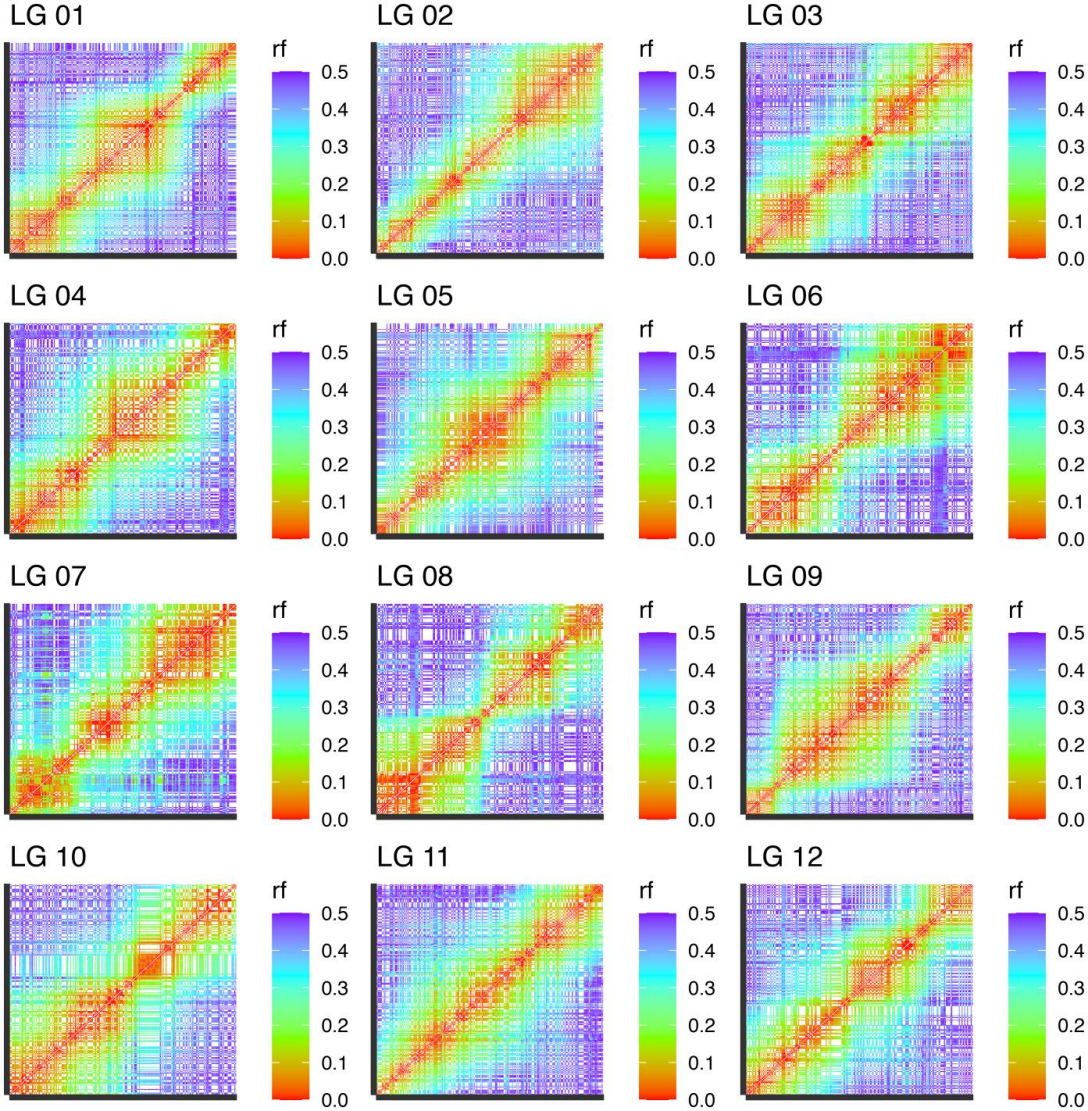
Recombination fraction (rf) matrices for each LG of *V. stamineum* biparental population. Lower recombination rate can be observed between adjacent markers in the diagonal, while higher recombination rate can be observed between distant markers in the upper left and lower right corners of the matrices.

**Fig. 3.**
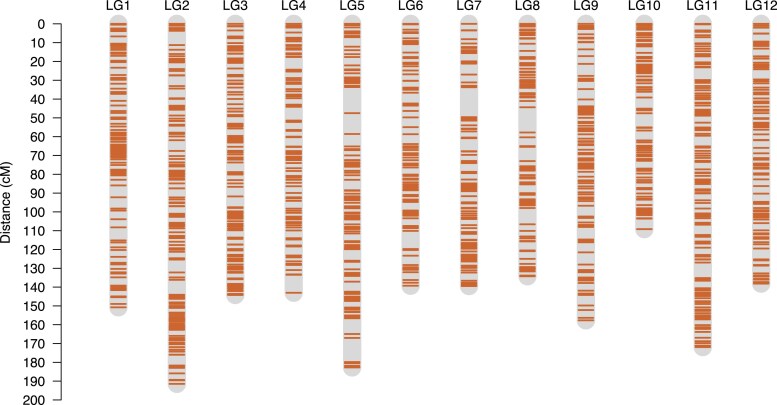
Distribution of SNP markers across the linkage groups (LGs) of *V. stamineum*, corresponding to the 12-base chromosome number of the species and spanning a genetic distance of 1,801.91 cM.

The recombination matrices of each LG were visually inspected for their marker ordering in iterative rounds of polishing, and necessary changes were applied as needed (Fig. 2). After manual curation to refine the LGs, the final map comprised 3,045 markers, with LG6 possessing the lowest number of markers at 206, and LG11 being the most populated with 311 markers (Fig. 3). Furthermore, the average interloci distance across the map was 0.59 cM, with the largest gap present in LG7, corresponding to 15.9 cM (Table 1). Finally, once the backbone of the *V. stamineum* genetic map was anchored with informative markers, the bins containing SNPs with redundant information were reincorporated into the genetic map, resulting in a final set of 3,797 SNPs.

**Table 1.** Summary of the linkage map built for *V. stamineum*.

Linkage group	# of SNPs	# SNPs w/redundants	Total length (cM)	Average Interloci distance (cM)
1	231	285	150.74	0.653
2	311	368	191.45	0.616
3	259	314	144.19	0.557
4	227	290	143	0.630
5	298	395	182.72	0.613
6	206	230	139.28	0.676
7	219	288	139.44	0.637
8	218	271	134.23	0.616
9	278	365	157.68	0.567
10	237	307	109.16	0.461
11	316	376	171.89	0.544
12	245	308	138.13	0.564
TOTAL	3045	3797	1801.91	-

### Phenotyping

**Fig. 4.**
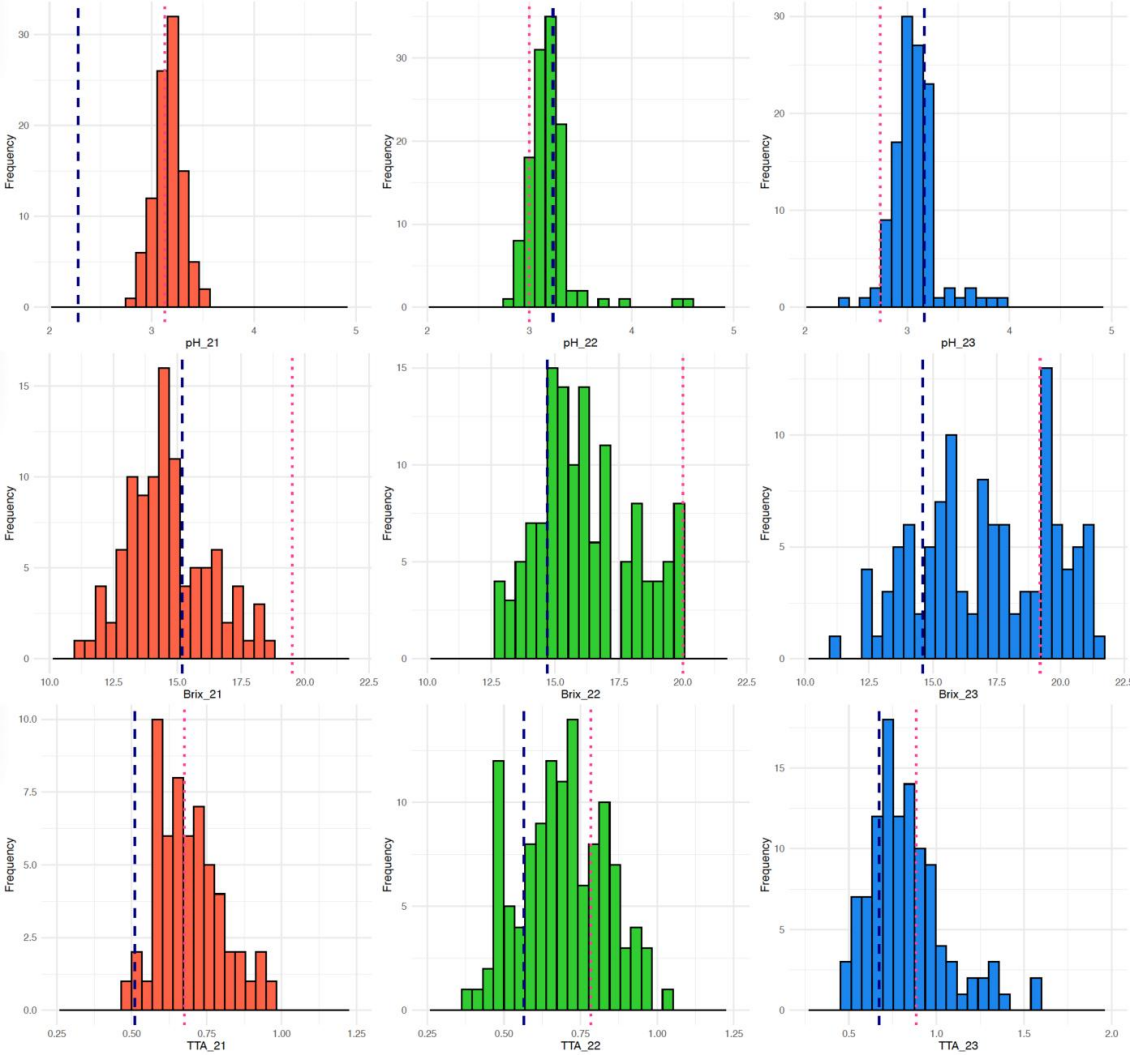
Phenotypic distribution of pH (top), °Brix (center), and TTA (bottom) for 3 yr of evaluations: 2021, 2022, and 2023, represented by the different colors. The dashed lines correspond to the “AP3” and “Crispsweet” parental genotypes.

The phenotypic assessments for fruit quality were conducted over a 3-yr period, and substantial phenotypic variability was observed across all 3 traits (Fig. 4). Interestingly, in 2021, the initial year of evaluation, the parent “AP3” exhibited lower pH values compared to the other parent “Crispsweet,” but this pattern was not replicated in the subsequent years of pH evaluation. For °Brix and TTA, the parental lines maintained consistent behavior throughout the 3 yr of phenotyping (Fig. 4).

Additionally, Spearman’s correlation analysis among the traits revealed moderate correlations between the years of phenotypic evaluation for a given trait (Supplementary Fig. 3), ranging from 0.09 for °Brix 2022 to 2023 to 0.66 for TTA 2021 to 2023. Another noteworthy observation is that pH and TTA demonstrated a moderate to strong negative correlation across all 3 yr of data collection, ranging from −0.16 to −0.50, while °Brix and TTA had a moderate positive correlation, especially for the 2022 and 2023 seasons (Supplementary Fig. 3).

### QTL mapping and estimation of genetic parameters

Low to moderate heritability was found for °Brix, TTA, and pH corresponding to 0.35, 0.28, and 0.23, respectively. In addition, the fixed year effect was significant for all traits (*P* < 000.1), while random genotype × year interaction effect was not significant for any trait.

QTL analysis was conducted separately for each trait and year of evaluation, with significance thresholds determined through 1,000 permutations. In total, 20 QTL were identified across the 3 traits and evaluation years. Interestingly, most QTL showed both significant additive effects (LOD ≥ 0.83) in at least 1 parent and notable dominance effects, suggesting that nonadditive genetic contributions may play an important role in the expression of these traits (Table 2).

**Table 2.** Summary of the QTL characterized for pH, TTA, and °Brix in *V. stamineum* biparental population over 3 yr of phenotypic evaluations.

QTL	LG	Position (cM)	Window Size	R2	Additive effect for “AP3”	LOD1	Additive effect for “Crispsweet”	LOD2	Dominance effect	LOD3
pH_2021	4	109.87	3,114,984	22.99	−0.03	2.89	0.03	2.57	0.06	6.90
pH_2021	12	123.00	4,279,369	14.38	0.06	7.35	−0.04	3.24	−0.05	4.57
pH_2023	1	58.52	1,488,271	7.95	0.05	1.46	0.09	3.61	−0.07	4.11
Brix_2021	1	133.00	2,794,979	10.28	0.13	0.24	0.09	0.11	0.77	7.50
Brix_2021	12	16.00	5,472,176	12.63	−0.57	4.57	0.15	0.37	−0.36	1.85
Brix_2022	1	66.41	5,562,306	8.57	−0.05	0.05	0.16	0.44	−0.71	6.80
Brix_2022	2	132.22	3,867,827	10.15	−0.71	7.70	0.19	0.63	0.41	2.78
Brix_2022	8	65.41	6,895,587	11.86	−0.45	2.97	0.61	5.94	−0.34	1.76
Brix_2022	9	72.77	2,982,708	5.81	0.45	3.30	0.38	2.63	−0.02	0.01
Brix_2023	9	62.77	525,163	9.97	0.42	1.14	0.06	0.03	0.93	5.68
Brix_2023	10	49.00	5,103,193	10.91	0.19	0.30	−0.95	5.86	0.79	4.24
TTA_2021	7	53.95	1,218,996	5.01	0.04	4.78	−0.04	3.98	−0.03	3.14
TTA_2021	8	60.35	4,136,986	3.97	−0.03	2.16	0.02	1.67	0.05	5.18
TTA_2022	4	24.38	5,946,487	18.18	−0.02	1.35	−0.07	11.41	0.01	0.24
TTA_2022	8	82.23	9,283,188	5.13	−0.04	3.98	0.04	4.19	0.00	0.00
TTA_2022	11	113.25	70,200	10.49	−0.04	4.44	0.00	0.03	−0.04	4.33
TTA_2022	12	74.29	9,949,092	10.32	−0.03	2.78	−0.03	1.59	0.03	2.55
TTA_2023	3	109.78	1,691,992	19.92	−0.09	7.63	0.02	0.55	−0.05	3.06
TTA_2023	9	65.00	5,427,379	13.27	0.00	0.00	−0.02	0.22	0.09	7.77
TTA_2023	9	21.30	1,109,304	9.92	0.03	0.65	−0.01	0.16	−0.08	5.74

Threshold for QTL detection was calculated with 1,000 permutations for each phenotype dataset individually considering a α = 0.05.

LOD ^1,2,3^ corresponds to the additive effect of “AP3,” “Crispsweet” parents, and dominance effect, respectively.

**Fig. 5.**
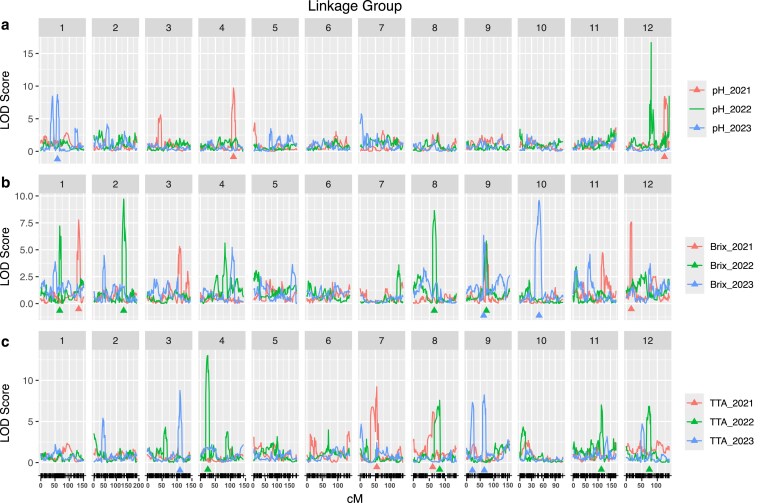
QTL mapping results for fruit quality traits across 3 yr of phenotypic evaluation (2021, 2022, and 2023) are depicted, with the significant loci indicated by triangles at the bottom of the plot. The color coding represents the different years. The significance threshold was determined through 1,000 permutations for each dataset individually, using an α = 0.05. a) The QTL analysis for pH, b) for Brix, and c) for TTA.

For pH, 3 QTL were detected in the 2021 and 2023 phenotypic assessments, while no significant peaks were found in 2022 (Fig. 5). In 2021, QTL on LG4 and LG12 accounted for approximately 23% and 14% of the phenotypic variation, respectively. In 2023, a single QTL on LG1 had a broad-sense heritability of about 8%.

Despite the moderate correlation between pH and TTA, no overlapping QTL peaks were observed between the 2 traits. A total of 9 QTL were identified for TTA over the years (Fig. 5). In 2021, 2 QTL on LG7 and LG8 explained 5.01% and 3.97% of the phenotypic variation, respectively. Interestingly the QTL in LG8 was also detected in the 2022 dataset, explaining approximately 5% of the phenotypic variation. In addition, in 2022, 3 other QTL on LG4, LG11, and LG12 accounted for approximately 12%, 10%, and 10% of the phenotypic variation, respectively. In 2023, a QTL on LG3 explained about 20% of the phenotypic variability, while 2 QTL on LG9 explained 13% and 9%, respectively.

The °Brix also showed a consistent QTL peak across multiple years of evaluation (Fig. 5). In 2021, 2 peaks on LG1 and LG12 explained 10% and 12% of the phenotypic variability, respectively. In 2022, significant peaks were found on LG1, LG2, LG8, and LG9, accounting for approximately 8%, 10%, 11%, and 5% of the phenotypic variability, respectively. In 2023, the same peak on LG9 as observed in 2022 explained about 10% of the phenotypic variability, while the second significant peak in LG10 explained 11% of the phenotypic variability.

## Discussion

### Phenotypic variability in fruit quality traits

This study aimed to assess the variability in fruit quality traits, including pH, TTA, and °Brix, within a biparental population of *V. stamineum* to better elucidate their genetic architecture. Among the *Vaccinium* wild species, *V. stamineum* is particularly notable for its inherently high levels of soluble sugars compared to other uncultivated species (Ballington et al. 1984). Previous reports have found that *V. stamineum* exhibits the highest average soluble solid content, even exceeding that of cultivated blueberry species like highbush blueberry (*V. corymbosum*) and rabitteye (*Vaccinium virgatum*) (Ballington et al. 1984). In the population of this study, the soluble solid content in *V. stamineum* ranged from 11.1 and 22.6, while in southern highbush blueberry breeding population, a range of 6.8 to 17.9 has been reported (Ferrão et al. 2018).

The variability of fruit quality parameters in this *V. stamineum* population followed a nearly normal distribution for °Brix and TTA, and a slightly skewed curve for TTA, reflecting the quantitative nature of these traits. The parental lines' profiles for °Brix and TTA followed consistent trends over the 3-yr evaluation period, while pH was more variable. The overall variation of the population across years was also high, considering the low to moderate correlations between the years of phenotypic evaluation. Previous research in 5 blueberry cultivars highlighted the impact of different environments and growing seasons on fruit quality parameters, such as TTA and soluble solids (Redpath et al. 2021; Mengist et al. 2022a, 2022b). Moreover, studying *V. stamineum* also poses an extra challenge, as its berries detach and drop as they ripen, making it difficult to predict and standardize the timing of berry collection. This variability in berry maturity can also introduce noise into the phenotypic dataset for fruit quality measurements.

### Probe design and genotyping

At the time that this study was designed, there was no reference genome available for *V. stamineum*, and probes designed for other *Vaccinium* species were used for genotyping the population. Once a genome became available (Matsumoto et al. 2025), we were able to successfully align 70.75% of the probes to the *V. stamineum* reference genome. Interestingly, although the genomes are highly collinear and syntenic to each other, a total of 1,755 probes did not have a corresponding match to the *V. stamineum* genome and, out of the remaining probes, 1,281 did not yield any polymorphic SNP markers in this population, leaving a set of 2,964 probes carrying useful genomic information for the following analysis. Therefore, more complex variants are to be expected between the genomes of *V. corymbosum* and *V. stamineum* in future comparative analyses.

### Linkage map construction and QTL identification

The genetic map presented in this study utilized only SNPs. The variant calling process was performed using strict parameters to ensure the reliability of the markers, minimizing the impact of potential sequencing errors or variant miscalls. While linkage maps have been previously reported for the cultivated highbush blueberry species, *V. corymbosum* (Cappai et al. 2020; Mengist et al. 2021, 2022a, 2022b; Montanari et al. 2022) and pseudo-backcross with *Vaccinium darrowii* (Qi et al. 2021), in this study, we generated the first genetic map constructed for the wild species *V. stamineum*. This map can serve as a valuable resource for further investigations of other traits, supporting future efforts to introduce this wild species into blueberry breeding.

Although the heritability estimates for fruit quality traits in *V. stamineum* were moderately low in this study, similar trends have been reported in *V. corymbosum*. Cellon et al. (2018) reported heritability estimates of 0.33 for soluble solids and 0.36 for pH. Likewise, de Bem Oliveira et al. (2019) found heritability values of 0.25 for both traits.

Previous research on *V. corymbosum* has identified QTL linked to fruit quality traits (Ferrão et al. 2018; Mengist et al. 2021). In this study, we obtained novel QTL as well as some that share similar genomic positions to what was previously reported in the literature. It is noteworthy that, despite being different species of the same genus, the *V. stamineum* genome shares the same base chromosome number as *V. corymbosum*, and these genomes exhibit a high degree of synteny (Matsumoto et al. 2025 ). Whole genome sequence alignments between the species showed that the QTL identified for TTA in 2023 on LG3 aligns with the QTL region found in 2 independent populations of *V. corymbosum* (Mengist et al. 2021, 2022a, 2022b) (Supplementary Table 1). Additionally, 2 QTL for °Brix have been reported on chromosome 8 and chromosome 10 of *V. corymbosum* (Mengist et al. 2022a, 2022b), which also overlaps with the QTL from this study in *V. stamineum* (Supplementary Table 1). Lastly, the QTL for pH in LG4, also has been reported in a *V. corymbosum* population in a corresponding genomic region (Supplementary Table 1). Overall, the QTL found in *V. corymbosum* populations had higher heritability than those identified in this study, which could be explained by the extensive phenotypic plasticity observed in *V. stamineum* and/or challenges in sampling berries at the consistent maturity stage.

Interestingly, a QTL for °Brix on LG9 and for TTA on LG8 were consistent for 2 yr of evaluation. To the best of our knowledge, this QTL has not been previously reported in blueberry, suggesting that the incorporation of this wild species, *V. stamineum*, into the blueberry breeding program may potentially expand the genetic diversity for improving sugar content, which is a key fruit quality trait for consumer liking.

In summary, this study represents a significant step toward integrating *V. stamineum* into the blueberry breeding pipeline by providing the first genetic map for this wild relative. The identification of a consistent QTL for °Brix on LG 09 highlights its potential to contribute valuable traits, such as enhanced sugar content, to cultivated blueberries. Furthermore, this research lays the groundwork for understanding the genetic diversity within the *Polycodium* section of *Vaccinium*, serving as an initial framework for the potential domestication of this species and its utilization in future breeding efforts.

## Supplementary Material

jkaf263_Supplementary_Data

## Data Availability

The raw genotypic data has been submitted to NCBI Sequence Read Archive (SRA) under the BioProject number PRJNA1354483. In addition, data was submitted to GSA Figshare portal (https://doi.org/10.25387/g3.29944349), including the following: **PhenoData_vstamineum.csv**: 3 yr of phenotypic data **genotypic_data_SNP_Vstamineum.csv**: SNP matrix **GeneticMap_Vstamineum.map**: genetic map (cM) **GeneticMap_Vstamineum.RData.gz**: R data exported from OneMap **SNPCalling_stamineum_hap1.vcf**: VCF file **vstamineum_rawdata.tar.gz:** raw fastq data Supplemental material available at *G3* online.
