# Supplementary material for: High-density linkage map and QTL analyses for fruit quality traits in the wild blueberry relative Vaccinium stamineum
Source: G3 (Bethesda). 2025 Nov 12;16(1):jkaf263. doi: 10.1093/g3journal/jkaf263 (PMC12774586; doi:10.1093/g3journal/jkaf263)
Supplement: jkaf263_Supplementary_Data [file jkaf263_supplementary_data.zip › Supplementary_Figure_1_G3-2025-406042.pdf]

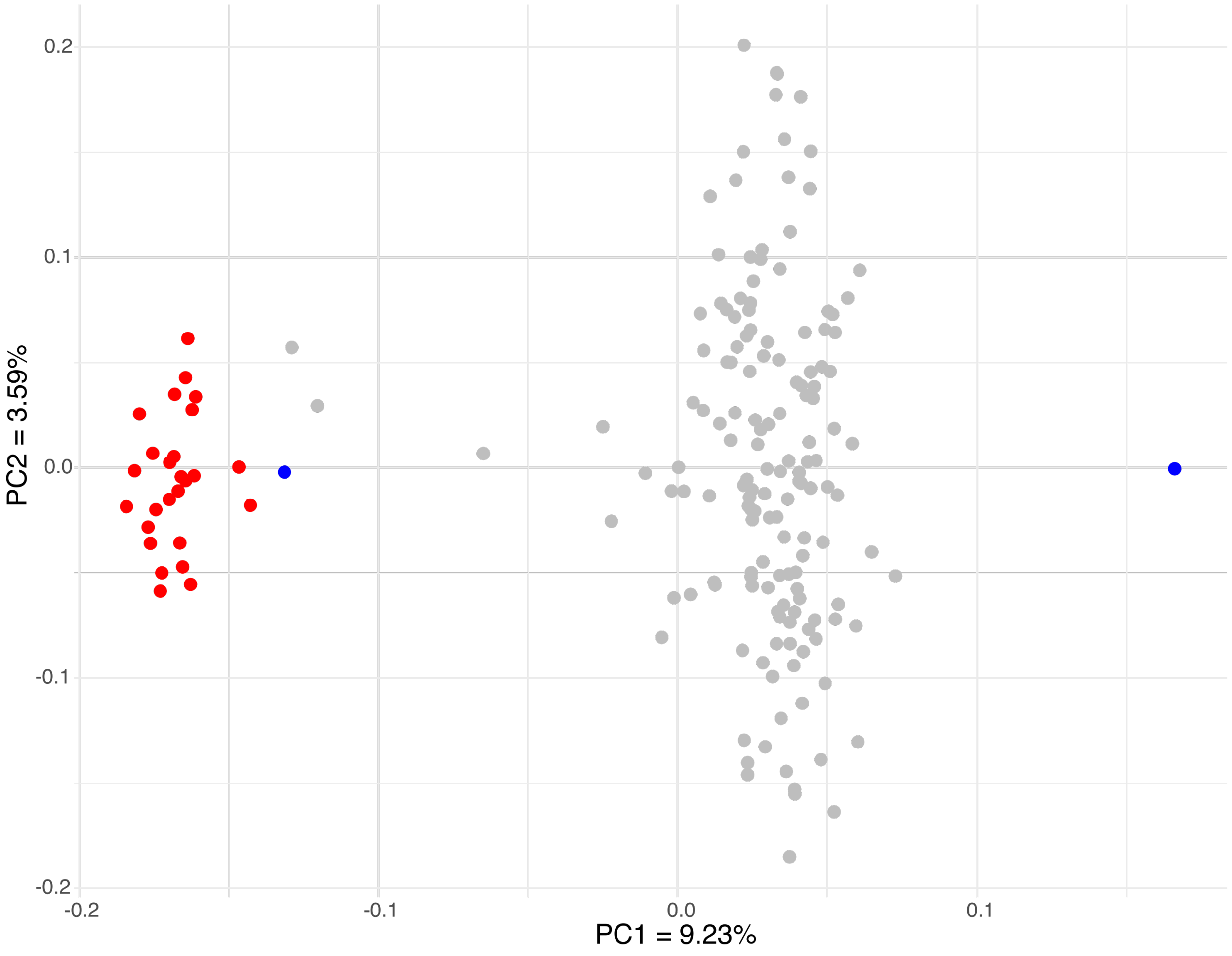

Supplementary Figure 1. Principal component analysis (PCA) using the SNP dataset from the biparental population of *V. stamineum* 'AP3' x 'CrispSweet'. The blue points represent the parental lines, while the red points depict individuals that were excluded from the analysis based on this clustering analysis.
