# Supplementary material for: High-density linkage map and QTL analyses for fruit quality traits in the wild blueberry relative Vaccinium stamineum
Source: G3 (Bethesda). 2025 Nov 12;16(1):jkaf263. doi: 10.1093/g3journal/jkaf263 (PMC12774586; doi:10.1093/g3journal/jkaf263)
Supplement: jkaf263_Supplementary_Data [file jkaf263_supplementary_data.zip › Supplementary_Figure_2_G3-2025-406042.pdf]

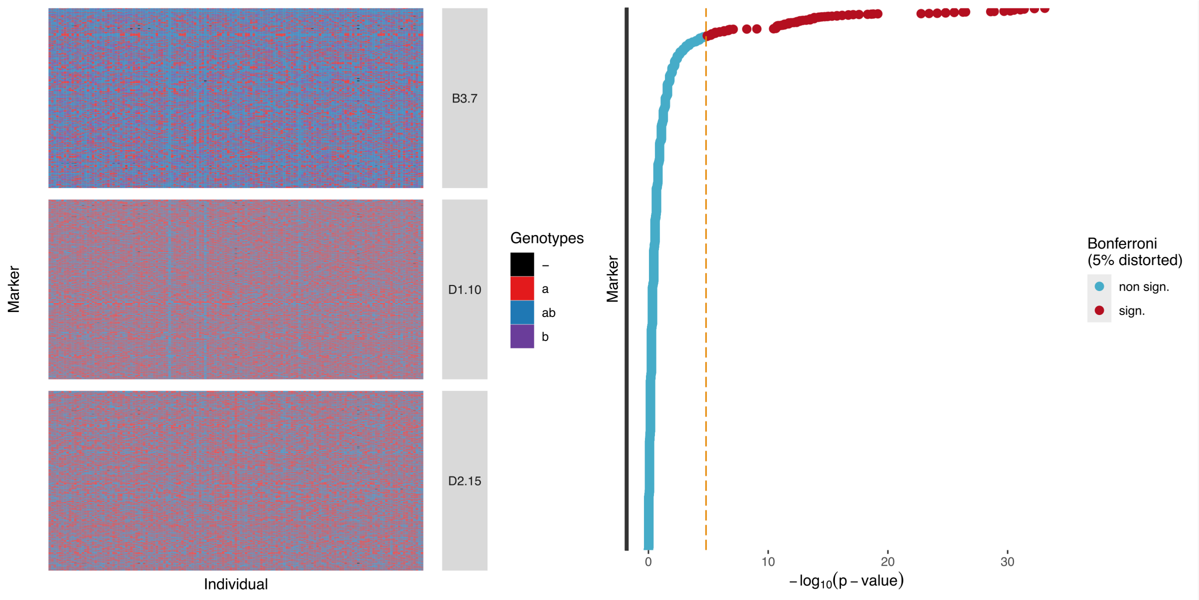

Supplementary Figure 2. Segregation patterns of biallelic SNP markers in the *V. stamineum* biparental population. On the left, progeny individual genotype for each marker divided into the three marker segregation classes: B3.7 - *ab* x *ab*; D1.10 - *ab* x *aa*; and D2.15 – *aa* x *ab*. On the right, a segregation distortion test to remove marker that do not follow the expected segregation ratios.
